# Mesoporous Bioactive Glass Functionalized 3D Ti-6Al-4V Scaffolds with Improved Surface Bioactivity

**DOI:** 10.3390/ma10111244

**Published:** 2017-10-27

**Authors:** Xiaotong Ye, Sander Leeflang, Chengtie Wu, Jiang Chang, Jie Zhou, Zhiguang Huan

**Affiliations:** 1 State Key Laboratory of High Performance Ceramics and Superfine Microstructure, Shanghai Institute of Ceramics, Chinese Academy of Sciences, 1295 Dingxi Road, Shanghai 200050, China; yexiaotongscu@foxmail.com (X.Y.); chengtiewu@mail.sic.ac.cn (C.W.); jchang@mail.sic.ac.cn (J.C.); 2 University of Chinese Academy of Sciences, No.19(A), Yuquan Road, Shijingshan District, Beijing 100049, China; 3 Department of Biomechanical Engineering, Delft University of Technology, Mekelweg 2, 2628 CD Delft, The Netherlands; M.A.Leeflang@tudelft.nl

**Keywords:** selective laser melting, titanium, scaffold, mesoporous bioactive glass, spin coating, in vitro bioactivity

## Abstract

Porous Ti-6Al-4V scaffolds fabricated by means of selective laser melting (SLM), having controllable geometrical features and preferable mechanical properties, have been developed as a class of biomaterials that hold promising potential for bone repair. However, the inherent bio-inertness of the Ti-6Al-4V alloy as the matrix of the scaffolds results in a lack in the ability to stimulate bone ingrowth and regeneration. The aim of the present study was to develop a bioactive coating on the struts of SLM Ti-6Al-4V scaffolds in order to add the desired surface osteogenesis ability. Mesoporous bioactive glasses (MBGs) coating was applied on the strut surfaces of the SLM Ti-6Al-4V scaffolds through spin coating, followed by a heat treatment. It was found that the coating could maintain the characteristic mesoporous structure and chemical composition of MBG, and establish good interfacial adhesion to the Ti-6Al-4V substrate. The compressive strength and pore interconnectivity of the scaffolds were not affected by the coating. Moreover, the results obtained from in vitro cell culture experiments demonstrated that the attachment, proliferation, and differentiation of human bone marrow stromal cells (hBMSCs) on the MBG-coated Ti-6Al-4V scaffolds were improved as compared with those on the conventional bioactive glass (BG)-coated Ti-6Al-4V scaffolds and bare-metal Ti-6Al-4V scaffolds. Our results demonstrated that the MBG coating by using the spinning coating method could be an effective approach to achieving enhanced surface biofunctionalization for SLM Ti-6Al-4V scaffolds.

## 1. Introduction

Three-dimensional (3D) Ti alloy-based scaffolds fabricated by means of selective laser melting (SLM) have attracted extensive interest in the field of orthopedics [1]. As compared with conventionally fabricated scaffolds that typically contain a vast number of randomly-shaped pores, SLM Ti alloy-based scaffolds have controllable 3D hierarchical porous structures [2,3,4], which not only ensures pore interconnectivity that is essential for cell ingrowth and nutrient transport, but also allows for the modulation of mechanical properties, especially Young’s modulus, to minimize stress shielding [5,6,7,8]. However, due to the inherent bio-inertness of titanium alloys, titanium alloy scaffolds can barely promote bone regeneration, which may significantly hinder their applications in certain orthopedic treatments, for example, in the repair of segmental bone defect [9].

The application of a bioactive coating on the strut surfaces of titanium alloy scaffolds has been proven to be an efficient strategy to improve the surface bioactivity of the metallic substrate [10,11]. For example, hydroxyapatite and bioactive glass coatings have been applied on the strut surfaces of titanium alloy scaffolds, which lead to changes in surface chemical composition and, thus, improved osteointegration and osteogenesis [12,13,14]. In fact, besides the chemical composition, the nanostructure of the surface plays an important role in promoting bone formation. It has been demonstrated that nanostructured surfaces can lead to rapid protein adsorption at the early stage of implantation, which subsequently mediates cellular responses, such as cell attachment and proliferation [15,16,17]. In recent years, mesoporous bioactive glasses (MBGs) have been developed as a new class of bioactive materials, and the most attractive feature is that they present an ordered mesoporous channel structure which, together with the chemical composition, leads to superior bioactivity to conventional bioactive glasses (BGs) [18,19,20,21,22,23]. In our previous study, MBG coating was successfully applied on the strut surfaces of calcium phosphate bioceramic scaffolds by using the spin coating technique. It was demonstrated that the presence of an MBG coating layer led to enhanced osteogenic differentiation of cells, as well as in vivo bone formation and bone growth into the scaffolds [22]. Based on this result, we hypothesized that MBG would also be an effective bioactive coating material to improve the osteogenic activity of Ti alloy scaffolds.

The aim of our present study was to apply MBG coating by means of spin coating on the strut surfaces of SLM Ti alloy scaffolds to improve their surface bioactivity. The surface morphology and chemical composition of the coating, as well as the effect of the coating on the mechanical strength and porosity of the scaffolds were investigated. In addition, cell adhesion, proliferation and osteogenesis differentiation on the MBG-coated SLM Ti-6Al-4V scaffolds were evaluated and compared with those on the BG-coated Ti-6Al-4V scaffolds and bare-metal Ti-6Al-4V scaffolds.

## 2. Results

### 2.1. Structural Characteristics of the Scaffolds

**Figure 1:**
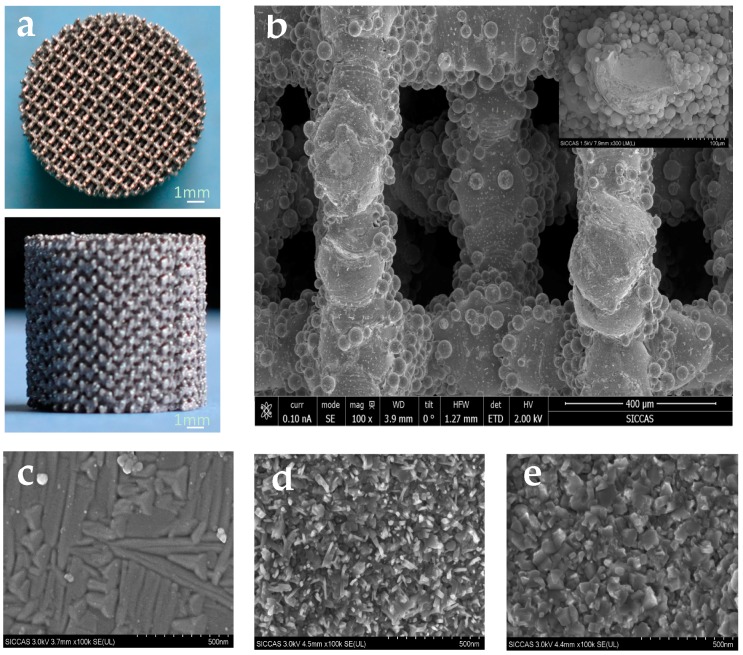
Overview of Ti-6Al-4V scaffolds (**a**); low-magnification view of scaffold struts showing macro pore sizes around 0.5 mm (**b**) together with an inserted high-magnification SEM image of the surface morphology of Ti-6Al-4V scaffold struts (**c**); BG-coated Ti-6Al-4V scaffold struts (**d**) and MBG-coated Ti-6Al-4V scaffold struts (**e**).

Figure 1a is a top view and a front view of the cylindrical Ti-6Al-4V scaffolds (d = 10 mm and h = 10 mm) fabricated by means of SLM machine (Realizer, SLM-125, Borchen, Germany). The low-magnification scanning electron microscopy (SEM; Hitachi S-4800, Tokyo, Japan) image of the scaffolds (Figure 1b) shows the surface morphology of struts. It is clear that the scaffolds, indeed, have a highly porous structure, and the diagonal length of the square pore is about 0.5 mm. The SEM image at a higher magnification (inset in Figure 1b) shows the presence of semi-attached Ti-6Al-4V microspheres on strut surfaces. The surface morphologies of Ti-6Al-4V, BG-coated Ti-6Al-4V and MBG-coated Ti-6Al-4V scaffold struts are presented in Figure 1c–e, respectively. It can be clearly seen that both the BG layer and MBG layer are composed of nanoscale particles.

**Figure 2:**
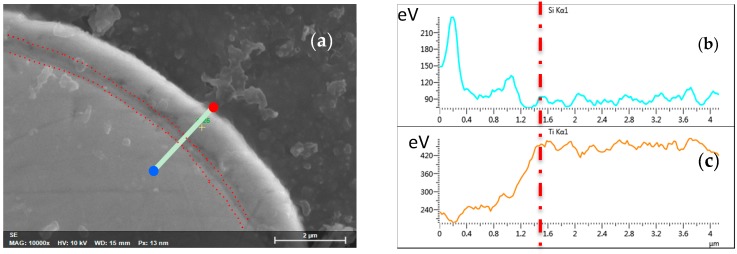
Cross-section morphology (**a**) of MBG-coated Ti-6Al-4V scaffold strut showing an interlayer and the MBG coating layer with a thickness of about 1 μm and EDS line scan analysis (from the red dot to the blue dot) showing interdiffusion of Si (**b**) and Ti (**c**) across the interlayer between the coating layer and substrate.

The cross-section morphology of the strut after MBG coating is shown in Figure 2a. The red dotted lines in the figure outline the matrix, SiO_2_ interlayer and MBG layer. The MBG coating layer is about 1 μm thick and it contains no pores, cracks, or other obvious defects at the interface between the coating layer, interlayer, and metallic substrate. Energy dispersive spectrometer (EDS; Hitachi S-4800, Tokyo, Japan) line scan analysis was performed from the coating to the substrate (the white line in Figure 2a, where the red dot and blue dot represent the starting point and ending point, respectively). From Figure 2b, it can be seen that interdiffusion of elements occurred between the coating and substrate, as evidenced by the decreases in Ti content from the substrate to the coating and the increases in Si content from the substrate to the coating. It was noticed that in Figure 2b the second maximum of the EDS profile of Si appeared at exactly the same spot as a maximum of the EDS profile of Ti, suggesting that Si and Ti atomic interdifussion took place between the coating and substrate [24].

**Figure 3:**
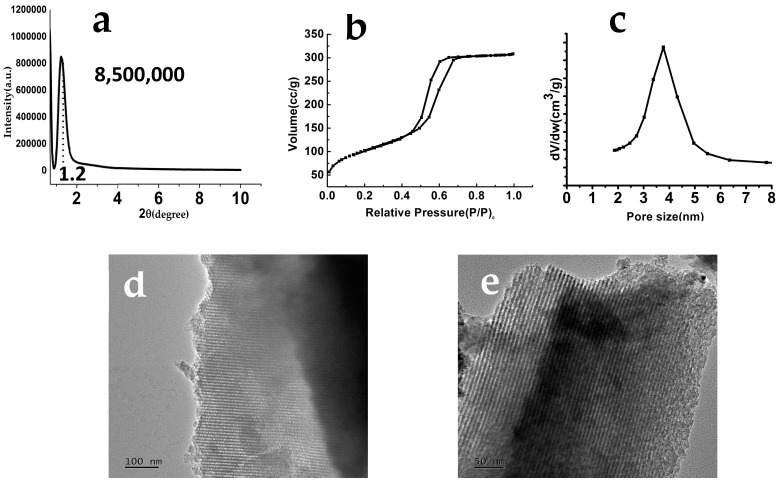
Grazing incidence X-ray diffraction (GIXRD) pattern of the MBG-coated Ti-6Al-4V scaffolds (**a**); nitrogen adsorption−desorption isotherms of the MBG powder (**b**); pore size distribution extracted from the N2 adsorption isotherms of the MBG powder (**c**); and TEM images for the MBG-coated Ti-6Al-4V scaffolds (**d**,**e**) with a well-ordered mesopore channel structure.

The grazing incidence X-ray diffraction (GIXRD; Geigerflex, Rigaku Co., Tokyo, Japan) pattern of the MBG-coated Ti-6Al-4V scaffolds confirmed the presence of the mesoporous structure, as evidenced by the diffraction peaks over a 2θ range of 0°–2° (Figure 3a). Nitrogen adsorption−desorption analysis of the MBG powder revealed a typical IV isotherm pattern with hysteresis loops of H1 type associated with the characteristic of cylindrical pores, in accordance with the p6mm mesostructure of MBG materials (Figure 3b). Figure 3c shows a pore size distribution extracted from the N_2_ adsorption isotherms of the MBG powder, indirectly confirming that the MBG coating had pore sizes around 4 nm, the calculated BET surface area was ~300 m^2^/g. Transmission electron microscopy (TEM; 2100F, JEOL, Tokyo, Japan) analysis clearly showed the typical well-ordered channels of MBG coated on Ti-6Al-4V scaffold strut surfaces (Figure 3d,e).

### 2.2. Mechanical Properties and Porosity of the Scaffolds

**Figure 4:**
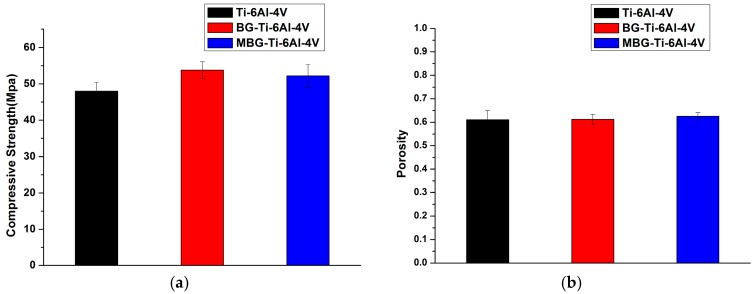
Compressive strengths (**a**) and porosity values (**b**) of the bare-metal Ti-6Al-4V scaffolds, BG-coated, and MBG-coated Ti-6Al-4V scaffolds.

Figure 4a shows the compressive strengths of the bare-metal Ti-6Al-4V, BG-coated and MBG-coated Ti-6Al-4V scaffolds. It was found that there were no statistically significant differences between the compressive strengths of the three groups.

A comparison in open porosity between the BG-coated and MBG-coated scaffolds and bare-metal scaffolds is shown in Figure 4b. The presence of the BG coating or MBG coating did not cause a significant change in open porosity.

### 2.3. Apatite Mineralization Ability of the MBG-Coated Ti-6Al-4V Scaffolds in SBF

**Figure 5:**
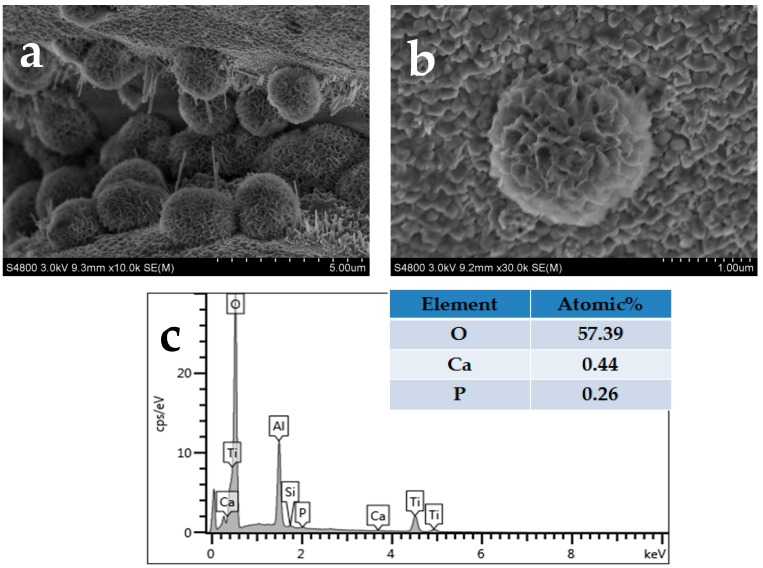
SEM surface morphology (**a**,**b**) of the MBG-coated Ti-6Al-4V scaffolds soaked in SBF for seven days and EDS analysis (**c**) of the flaky structure.

In vitro surface bioactivity of the MBG-coated scaffolds was evaluated in terms of hydroxyapatite (HA) mineralization during the immersion in simulated body fluid (SBF). Figure 5 shows the morphology of MBG-coated Ti-6Al-4V scaffold strut surface after the scaffolds were immersed in SBF for seven days. It can be seen that agglomerates of crystals with a flaky structure were formed on the surface, which is characteristic of HA. EDS analysis (Figure 5c) of the flaky structure revealed a Ca/P atomic ratio of 1.67.

### 2.4. Ion Release from the Scaffolds to the Tris-HCl Buffer Solution

**Figure 6:**
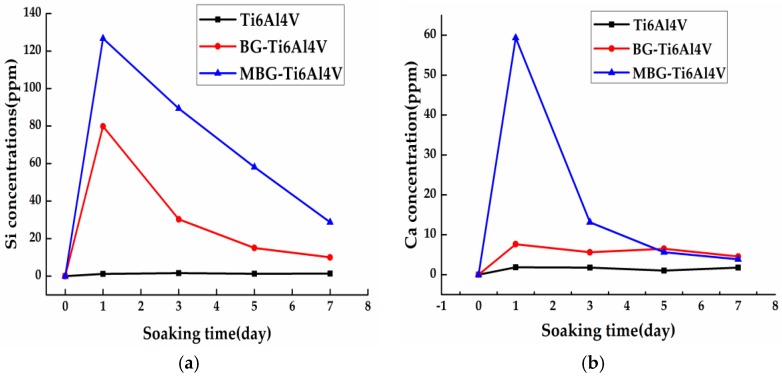
Ion concentrations of (**a**) Si and (**b**) Ca in the Tris-HCl solution after soaking of the scaffolds for one, three, five, and seven days.

Figure 6 shows the changes of the concentration of Ca and Si ions from the three groups of scaffolds in the Tris-HCl buffer solution after various soaking periods. The MBG-coated Ti-6Al-4V and BG-coated Ti-6Al-4V scaffolds released significant amounts of Si on day 1, and the amounts reduced in the following three, five, and seven days. The MBG-coated Ti-6Al-4V scaffolds released the largest amount of Ca on day 1 and the Ca ion release decreased abruptly, and then gradually, in the following days. For the bare-metal Ti-6Al-4V scaffolds and BG-coated Ti-6Al-4V scaffolds, however, the release profiles were very different; they largely remained flat from day 1 to day 7. It is worth noting that the MBG-coated Ti-6Al-4V scaffolds released the largest amounts of Ca and Si ions among the three groups, reaching 60 and 120 ppm on day 1, respectively.

### 2.5. In Vitro Osteogenesis of hBMSCs Cultured with the Scaffolds

#### 2.5.1. Cell Adhesion and Proliferation on the Scaffolds

**Figure 7:**
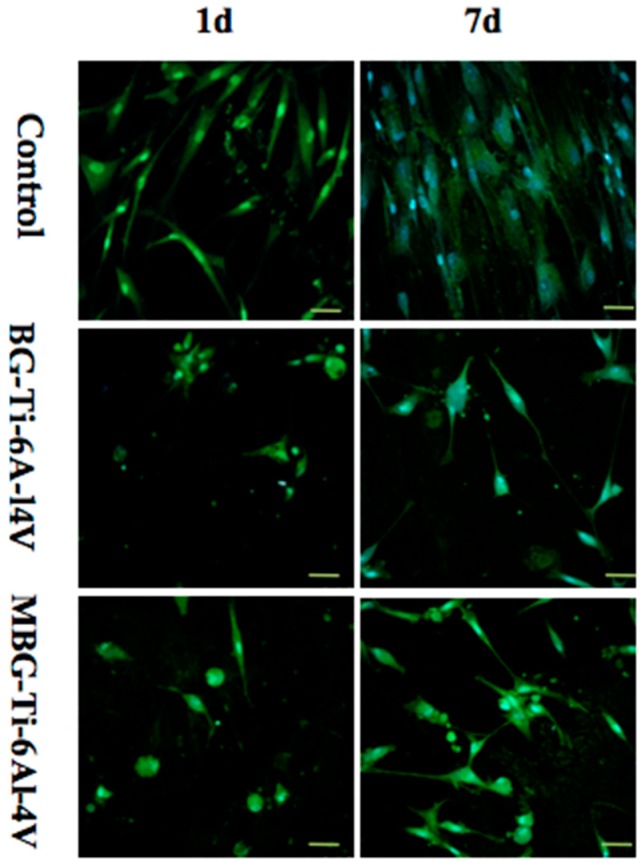
Confocal images showing the morphology and cytoskeleton of hBMSCs attached to the strut surfaces of the bare-metal Ti-6Al-4V, BG-coated Ti-6Al-4V and MBG-coated Ti-6Al-4V scaffolds at day 1 and day 7 (scale bar: 50 µm).

HBMSCs were fluorescently stained to investigate their adhesion behavior. Figure 7 shows confocal images of hBMSCs after culturing on strut surfaces for one day and seven days. It can be seen that the hBMSCs spread well and showed numerous filopodia on the strut surfaces of all the groups of the scaffolds. On day 7, the BG-coated Ti-6Al-4V and MBG-coated Ti-6Al-4V scaffolds showed distinct and well-defined microfilaments as well as cytoskeleton, and a more spreading and active morphology than those on day 1.

**Figure 8:**
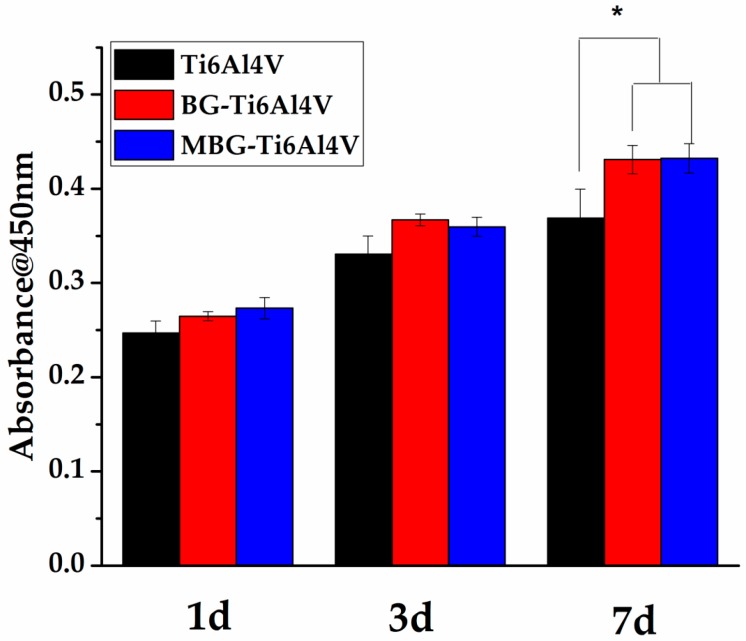
Viability of hBMSCs after culturing with the three groups of scaffolds for one, three, and seven days. (* *p* < 0.05, significant difference compared to the blank control).

HBMSCs were cultured on the three groups of the scaffolds for one, three, and seven days to investigate cell proliferation behavior. The proliferation of hBMSCs on the strut surfaces, determined by the CCK-8 assay, is shown in Figure 8. It can be seen that none of the groups showed cytotoxicity and there were no significant differences in cell proliferation rate between the three groups on day 1 or day 3. On day 7, however, the MBG-coated Ti-6Al-4V and BG-coated Ti-6Al-4V scaffold groups showed more pronounced cell proliferation than the bare-metal Ti-6Al-4V scaffold group.

#### 2.5.2. Osteogenic Differentiation of hBMSCs on the Scaffolds

**Figure 9:**
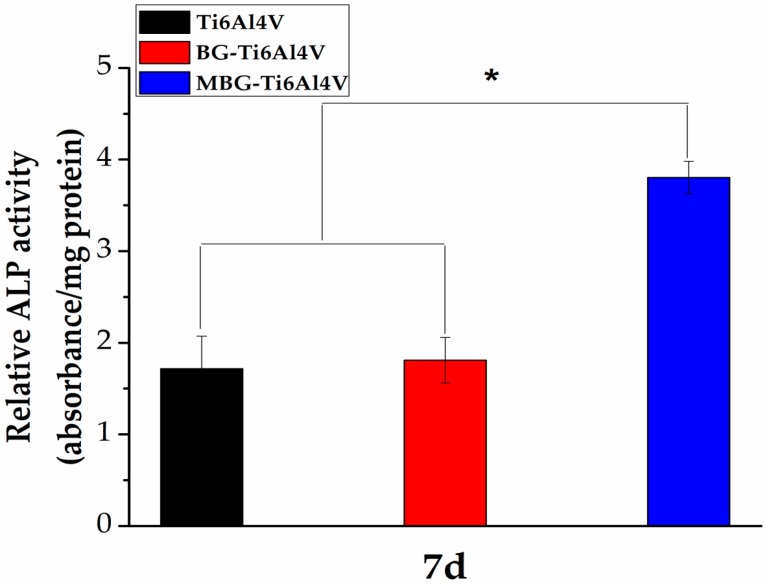
Osteogenesis-related gene expression ALP of hBMSCs after culturing with the three groups of scaffolds on day 7 (* *p* < 0.05, significant difference compared to the blank control).

From Figure 9, it can be seen that on day 7, as compared with the bare-metal Ti-6Al-4V scaffolds, the BG-coated Ti-6Al-4V scaffolds showed no significant difference in terms of ALP activity expression. However, the MBG-coated Ti-6Al-4V scaffolds showed the highest ALP activity as compared with the bare-metal Ti-6Al-4V and BG-coated Ti-6Al-4V scaffold groups, indicating the greatest potential of osteogenesis.

## 3. Discussion

It has been repeatedly demonstrated that Ti-6Al-4V scaffolds fabricated by means of SLM possess controllable geometrical features and preferable mechanical properties, both of which are desirable for orthopedic applications [2,4,6]. It is, however, well known that the strut surfaces of such scaffolds lack desirable features for biofunctionalization, such as bioactive elements and nanoscale cues to actively stimulate bone ingrowth and regeneration, when the scaffolds are implanted in the human body. Numerous studies have confirmed that surface chemical composition and nanostructures are the important factors affecting the biological effects of scaffold materials. Additionally, some studies have even confirmed that there is a synergistic effect between these two factors [15,16]. Therefore, in the present study, the strategy we adopted was to realize the multi-level combination of macroporosity, surface chemical composition and mesoporous structure by applying MBG coating on the strut surfaces of SLM Ti-6Al-4V scaffolds [25,26]. In our preliminary experiments, it was found that the heat treatment that was essential for the formation of mesoporous structure of MBG could lead to a detrimental interface reaction between the Ti-based substrate and MBG coating, thereby resulting in a destroyed microstructure of the MBG coating. In addition to the interface reaction, the difference in thermal expansion coefficient between the metal substrate and amorphous MBG coating might cause damage to the MBG coating during the heat treatment [27]. Therefore, in the present study, we designed and developed a SiO_2_ interlayer as a transition layer to ensure that the MBG coating maintained its structure and composition—an approach that has been used between the substrate and functional coating of other materials [28,29,30,31,32]. By using this approach, a 1 μm thick dense nano-structured MBG layer was uniformly deposited on the strut surfaces of the Ti-6Al-4V scaffolds. The MBG coating showed no obvious cracks and appeared to adhere well to the substrate through the interlayer. We deemed that the addition of the SiO_2_ interlayer could have promoted the adhesion between the amorphous MBG layer and the metal substrate. Obviously, a good interface strength is of vital importance for the scaffolds, especially for load-bearing applications, because if fracture and even exfoliation of the MBG layer occur, the internal structure of the scaffold would not be completely covered and as a result the bioactivity would not be uniform throughout the scaffold.

In this study, we found that the mesoporous structure and composition of MBG were well maintained and these characteristics would ensure the biological properties expected of mesoporous glass [33,34,35].

Mechanical strength and porosity are important mechanical and physical features of Ti alloy scaffolds, which represent the load bearing capacity and the opportunity of cell ingrowth, respectively. Therefore, it is of great importance that processing for scaffold surface bioactivation should not negatively affect these features. Our results demonstrated that the MBG coating did not markedly change the compressive strength and porosity of the original scaffolds, which means that the original mechanical performance of the Ti-6Al-4V scaffolds could be maintained. Considering the fact that the pore size and strut size were in the range of hundreds of micrometers whereas the thickness of the coating was merely one micrometer, the negligible decrease in porosity as a result of the addition of the coating layer on the strut surfaces would not cause significant changes to the biological performance of the scaffolds, except enhanced bioactivity. The uniform, thin coating layer on the struts of the scaffolds could be attributed to the spin coating process used. During the process, the centrifugal force could help the precursor solution evenly distribute inside the scaffolds and prevent the precursor from clogging [36,37].

In vitro bioactivity can be assessed by HA formed on the surface of the sample in SBF [18,38,39,40]. For pure titanium, there have been a number of studies, all demonstrating that the pure metal surface lacks the ability to induce HA deposition, which results in a lack of good bonding between the implant surface and the surrounding bone tissue [15,41]. Our results demonstrated that the MBG-coated Ti-6Al-4V scaffolds possessed a good apatite mineralization ability, which could be due to the excellent ability of mesoporous glass to induce apatite deposition on its surface [18,40]. The surface apatite mineralization of biomaterials is expected to contribute to the osteogenic activity of the materials. In our study, we found that hydroxyapatite was deposited on the MBG coating of the scaffolds after one day of soaking, and the amount of deposition increased significantly after seven days. Such a result is consistent with that reported in previous studies, which demonstrated that a large surface area of MBG promoted the deposition of Ca and P ions, which is essential for hydroxyapatite formation [42,43,44]. It is, thus, believed that the coating developed in this study can maintain the bioactive features of the MBG powder. Thus, the enhanced biological activity of the coating can be expected.

The ability to support cell attachment and proliferation is important for the surface biocompatibility of an implant [45]. As shown in our study, hBMSCs on the MBG-coated Ti-6Al-4V scaffolds showed a more spread morphology and a larger quantity than those on the bare-metal Ti-6Al-4V and BG-coated Ti-6Al-4V scaffolds, indicating the superior surface biocompatibility of the MBG-coated scaffolds. Moreover, we found that the MBG-coated Ti-6Al-4V scaffolds exhibited the highest ALP activity of hBMSCs, which is commonly considered to be a key marker of early-stage mineralization and osteogenic differentiation, indicating a stronger osteogenesis ability [46,47]. The observed differences in ALP activity may be due to ion release and mesoporous structures, as the superior ability of MBG to enhance osteogenic differentiation of hBMSCs is partly attributed to the more efficient release of Ca and Si ions, as compared with BG. The mesoporous structure is also an important contributor to promoting osteogenic differentiation, as it has been confirmed that the mesoporous structure can enhance protein adsorption [22,25,33,48]. Our results confirmed the superior bioactivity of the MBG-coated Ti-6Al-4V scaffolds to that of the BG-coated Ti-6Al-4V and bare-metal Ti-6Al-4V scaffolds, indicating that the MBG-coated scaffolds are worth further in vivo evaluation in terms of bone regeneration ability.

In conclusion, we have successfully applied MBG coating on the strut surfaces of the SLM Ti-6Al-4V scaffolds by means of spin coating. The characteristic mesoporous structure and chemical composition of MBG were maintained. The compressive strength, pore dimensions, and pore interconnectivity of the scaffolds were not compromised by adding a thin coating layer. Moreover, the results from the in vitro cell culture experiments demonstrated that the attachment, proliferation, and differentiation of hBMSCs on the MBG-coated Ti-6Al-4V scaffolds were much improved, as compared with those on the BG-coated and bare-metal Ti-6Al-4V scaffolds. Our results demonstrated that the developed MBG coating could be an effective approach to achieving enhanced surface bio-functionalization for SLM Ti-6Al-4V scaffolds.

## 4. Materials and Methods

### 4.1. Preparation of the MBG-Coated Ti-6Al-4V Scaffolds and Coating Precursor Solutions

A medical-grade Ti-6Al-4V powder (grade 23) supplied by Advanced Powders and Coatings (AP&C, Boisbriand, QC, Canada) with a median particle size of 31.6 μm and a spherical morphology was used to produce cylindrical scaffold samples. Its chemical composition is given in Table 1. Cylindrical scaffolds with a diameter of 10 mm and a height of 10 mm were 3D printed in the axial direction by using an SLM machine (Realizer, SLM-125, Borchen, Germany) with a YLM-400-AC ytterbium fiber laser (IPG Photonics Corporation, Oxford, MA, United States) under an inert atmosphere (argon) with an oxygen content below 0.2%. The layer thickness was 50 μm. The scaffolds had a diamond lattice structure with a strut thickness of 300 μm, corresponding to a porosity value of 68% (design value). No post-SLM processing was performed.

Prior to the application of MGB coating, a SiO_2_ interlayer was deposited on the strut surface with the intention to avoid a severe reaction between the substrate and MBG coating, which might lead to a changed chemical composition and destroyed mesoporous structure of MBG. A SiO_2_ precursor solution was prepared by hydrolyzing 20.1 g tetraethyl orthosilicate (TEOS) in 180 g ethanol solution, using 0.5 M HCl as catalyzer. An MBG precursor solution was prepared according to the procedure developed in a previous study [18]. First, 12 g of nonionic block copolymer EO20PO70EO20 (P123, Sigma-Aldrich, St. Louis, MO, USA ) was dissolved in 180 g ethanol and the solution was stirred to achieve clarification. Then, 20.1 g TEOS, 4.2 g Ca(NO_3_)_2_·4H_2_O, 2.19 g triethyl phosphate (TEP, 99.8%) and 3 g 0.5 M HCl were added to the ethanol P123 solution, TEOS, Ca(NO_3_)_2_·4H_2_O, TEP and HCl with AR grade were purchased from Sinopharm Chemical Reagent Co., Ltd, Shanghai, China. The Si/Ca/P molar ratio was set to be 80:15:5. The mixture solution was then stirred for 24 h. The obtained SiO_2_ precursor solution was deposited on the strut surfaces of the Ti-6Al-4V scaffolds by spin coating four times, and rotational speed was set at 500 rpm for the first 10 s and 2000 rpm for the following 20 s. Between the two coating runs, the scaffolds were kept in a fume hood for 8 h to allow the volatile components to evaporate. Then the MBG precursor solution was used to coat the scaffolds, following the same procedure. Dried gel was obtained by using the evaporation-induced self-assembly (EISA) method. Finally, the coated scaffolds were heated at a rate of 1 °C min^−1^ to 650 °C and held at 650 °C for 5 h to remove organic compounds and form the mesoporous structure. For comparison purposes, the conventional bioactive glass (BG) was applied to the strut surfaces of the Ti-6Al-4V scaffolds. The SiO_2_ precursor was the same as that applied to the BMG-coated scaffolds. In the BG precursor no P123 was added during preparation.

### 4.2. Surface Characterization of the MBG-Coated Ti-6Al-4V Scaffolds

Grazing incidence X-ray diffraction (GIXRD) was used to analyze MBG phase compositions. The surface morphology and the chemical composition changes from the MBG coating to the strut interior of the scaffolds were characterized by using scanning electron microscopy (SEM) and energy dispersive spectrometry (EDS), respectively. To evaluate the adhesion between the coating and substrate, the sample was cut into two pieces, embedded in resin, and then polished to expose the substrate-coating interface, which was then observed by using SEM. The mesoporous structure of the MBG coating was ascertained by using transmission electron microscopy (TEM). The Barrett-Emmett-Teller (BET) method was used to determine the pore type and calculate the specific surface area (S) of the coating.

### 4.3. Mechanical and Porosity Tests of the Scaffolds

The BG-coated and MBG-coated Ti-6Al-4V scaffolds and bare-metal Ti-6Al-4V scaffolds (d = 10 mm and h = 10 mm) were subjected to compression tests and porosity tests. The compression tests were carried out using a computer-controlled universal testing machine (Instron-5592, Boston, MA, USA) and the crosshead speed was set at 0.2 mm min^−1^. The porosity values of the coated and non-coated scaffolds were determined using a multi-function porosity and density tester (ET-320VP). For each group, six samples were used in order to ensure repeatability.

### 4.4. Immersion Tests of the Scaffolds

To evaluate the in vitro bone-like hydroxyapatite formation ability, the scaffolds were immersed in simulated body fluid (SBF) in a shaker at 37 °C. SBF was prepared according to the method described by Kokubo et al. [28]. All the chemical reagents involved in the preparation of SBF were purchased from Sinopharm Chemical Reagent Co., Ltd, Shanghai, China. The ratio of the volume of SBF to the weight of the sample was set at 50 mL/g, and the immersion medium in 50 mL centrifuge tube was refreshed once every two days. After one and seven days of immersion, the scaffolds were taken out of the SBF solution, gently rinsed with distilled water and dried at 60 °C overnight. The microstructure and chemical composition of the surface layer on the struts of the scaffolds were then characterized by using SEM and EDS, respectively.

The ion release behaviors of the Ti-6Al-4V, BG-coated Ti-6Al-4V and MBG-coated Ti-6Al-4V scaffolds were characterized by performing immersion tests in a Tris-HCl buffered solution in a shaker at 37 °C and the ratio of the volume of the Tris-HCl buffered solution volume to the weight of the scaffold was set at 4 mL/g. The soaking medium in 4 mL polyethylene bottle was refreshed once every two days. After one, three, five, and seven days of immersion, the soaking medium was collected, and the concentrations of Ca and Si ions in the collected solution were determined by using inductively-coupled plasma atomic emission spectrometry (ICP-AES) (Varian Co., Palo Alto, CA, USA). Three samples of each group in the immersion solution were tested.

### 4.5. In Vitro Biocompatibility and Osteogenic Ability of the Scaffolds

#### 4.5.1. Cell Adhesion and Proliferation

Human bone marrow stromal cells (hBMSCs) were purchased from Cyagen Biosciences. 1 × 10^4^ of hBMSCs (at passage 4) were added to the bare-metal Ti-6Al-4V, BG-coated Ti-6Al-4V and MBG-coated Ti-6Al-4V scaffolds (*n* = 3) in 48-well culture plates. The hBMSCs were incubated for one, three, five, and seven days in human mesenchymal stem cell basal medium supplemented with 10% human mesenchymal stem cell-qualified fetal bovine serum, 5% penicillin-streptomycin and 5% glutamine (Cyagen Biosciences, Santa Clara, CA, USA) under a 5% CO_2_ atmosphere at 37 °C. Confocal laser scanning microscopy (CLSM, Leica TCS SP8, Wetzlar, Germany) was used to observe the morphology of hBMSCs cultured on the three groups. Cellular samples were fixed with 2.5% glutaraldehyde for 20 min, followed by washing three times to remove excess glutaraldehyde, then the fixed cell cytoskeletons were stained with fluorescein isothiocyanate labeled phalloidin (FITC, Sigma-Aldrich, St. Louis, MO, USA), followed by washing three times with PBS to remove the nonspecific background, and cell nuclei were stained with 4′,6-diamidino-2-phenylindole (DAPI, Sigma-Aldrich, USA), following the same procedure. Argon laser line of 405 nm (DAPI channel, blue) and 488 nm (FITC channel, green) were used to capture the image. For cell proliferation assays, the relative cell proliferation rate was studied using Cell Counting Kit-8 (CCK-8, Dojindo, Rockville, MD, USA). Briefly, at each time point, samples were refreshed with a 10% CCK-8 and 90% hBMSCs basal medium mixed solution and incubated at 37 °C for 2 h. Then, 100 μL of the reaction solution was transferred into a new 96-well plate and the optical density was measured at 450 nm by a microplate reader (Epoch Microplate Spectrophotometer, BioTek Instruments, Winooski, VT, USA).

#### 4.5.2. Alkaline Phosphate (ALP) Activity Tests

To determine the early differentiation of hBMSCs stimulated by the three groups of the scaffolds, hBMSCs (1 × 10^4^ cells/well) were seeded on the bare-metal Ti-6Al-4V, BG-coated Ti-6Al-4V and MBG-coated Ti-6Al-4V scaffolds (*n* = 3). At day 7, the cells were permeabilized in 0.1% Triton X-100 for 10 min and then washed with PBS for three times. The lysates were centrifuged at 14,000 rpm for 15 min. 50 mL of supernatant was mixed with 150 mL of ALP assay working solution according to the manufacturer’s protocol (QuantiChromt Alkaline Phosphatase Assay Kit, BioAssay Systems, Hayward, CA, USA). The OD values were measured at 405 nm using a spectrophotometer. The relative ALP activity was expressed as the changed OD value divided by the reaction time and the total protein content was measured by the bicinchoninic acid protein assay kit (BCA, Sigma-Aldrich, USA).

### 4.6. Statistical Analysis

The experimental data were averaged and expressed as the mean ± standard deviation. Significant differences between different films were determined using the *t*-test, for which *p* < 0.05 was considered statistically significant.

## Figures and Tables

**Table 1:** Chemical composition of the powder used in this research (by weight percent).

Element	C	O	N	H	Fe	Al	V	Ti
Content (wt %)	0.02	0.10	0.02	0.0017	0.19	6.4	4.0	Balance
